# “Food” and “non-food” self-regulation in childhood: a review and reciprocal analysis

**DOI:** 10.1186/s12966-020-00928-5

**Published:** 2020-03-10

**Authors:** Catherine G. Russell, Alan Russell

**Affiliations:** 1 Faculty of Health, School of Exercise and Nutrition Sciences, Deakin University, 221 Burwood Highway, Burwood, VIC 3125 Australia; 2 College of Education, Psychology and Social Work, Flinders University, Sturt Rd, Bedford Park, South Australia 5042 Australia

**Keywords:** Appetite regulation, Energy intake, Executive function, Homeostasis, Self-regulation, Disinhibited eating, Effortful control, Inhibitory control, Top-down, Bottom-up

## Abstract

**Background:**

In developmental science, there is an extensive literature on non-food related self-regulation in childhood, where several domains relating to emotions, actions and cognitions have been identified. There is now growing attention to food related self-regulation in childhood, especially difficulties with ASR, and the consequences for weight gain and adiposity. The aim of this narrative review was to conduct a reciprocal analysis of self-regulation in the food and non-food domains in childhood (referred to as appetite self-regulation (ASR) and general self-regulation (GSR) respectively). The focus was on commonalities and differences in key concepts and underpinning processes.

**Methods:**

Databases and major journals were searched using terms such as self-regulation, appetite self-regulation, or self-regulation of energy intake, together with associated constructs (e.g., Executive Function, Effortful Control, delay-of-gratification). This was followed by backward and forward snowballing.

**Results and discussion:**

The scholarship on GSR in childhood has had a focus on the role of the cognitively-oriented Executive Function (EF), the temperamentally-based Effortful Control (EC) and the recursive interplay between bottom-up (reactive, emotion driven, approach or avoidance) and top-down (cognitive, conscious decision-making) processes. “Hot” and “cool/cold” EF and self-regulation situations have been distinguished. There were some parallels between GSR and ASR in these areas, but uncertainty about the contribution of EF and EC to ASR in young children. Possible differences between the contribution to ASR-related outcomes of delay-of-gratification in food and non-food tasks were apparent. Unique elements of ASR were identified; associated with psychological, biological and neurological responses to food and bottom-up processes. A diverse number of situations or elements connected to ASR exist: for example, energy balance homeostasis, caloric compensation, hunger regulation, satiation, satiety, energy density of food, eating in the absence of hunger, emotional eating, etc.

**Conclusions:**

Self-regulation in food and non-food domains are amenable to a reciprocal analysis. We argue that self-regulation of appetite should be added as a domain under the umbrella of self-regulation in childhood along with the other non-food related domains. This could lead to a broader understanding of self-regulation in childhood, and generate novel lines of enquiry.

## Background

Self-regulation is important for children’s healthy functioning and development, as shown by its associations with a wide range of developmental outcomes, including behavioral, social, emotional and academic adjustment [1–8], school readiness [9, 10] positive health outcomes [11] and overweight/obesity [7, 12–16]. Self-regulation has been identified as a central aspect of development in the early years [4, 8, 11, 17–19].

To clarify different forms of self-regulation, Saltzman and colleagues [20] and Anderson and Keim [21] drew a separation between food and non-food related self-regulation in childhood and referred to non-food related self-regulation as “general” self-regulation. In the childhood developmental science literature, Saltzman et al. and Anderson and Keim’s “general” self-regulation is simply referred to as self-regulation. It has received extensive research and theoretical attention [4, 18, 22–25]. In this literature, self-regulation has been conceived as an umbrella term, with the development of self-regulation occurring across a number of domains or levels of functioning including physiological arousal, attentional engagement and disengagement, emotional regulation, behavioral regulation, and executive cognitive control processes [3, 5, 22, 23], but not appetite. For present purposes, we refer to the “self-regulation” from developmental science as general self-regulation (GSR).

Scholarship on food-related self-regulation in childhood has emerged rapidly in recent years. It has included attention to self-regulation of energy intake (SREI) [26–29], and more generally to appetite self-regulation (ASR) or self-regulation of eating [13, 20, 30–34] where self-regulation difficulties have been repeatedly associated with poor dietary intakes and weight status in children.

ASR is a general construct and incorporates the roles of both hunger and satiety in prompting and stopping energy intake [20]. It includes not only energy intake, but diet quality (e.g., selection of healthy or unhealthy food), and is closely linked with energy expenditure. ASR covers the positive aspects of regulation, but also the inverse in terms of the disruption of ASR, in the form of disinhibited eating and related concepts. The significance of food related self-regulation is recognized by claims [35–37] that healthy eating and food decisions require effortful and goal-directed self-regulation. At the same time, self-regulation is only one of many factors that contribute to dietary intake and the development of overweight and obesity (OW/OB) [16, 31, 38–42]. Nevertheless, difficulty with appetite self-regulation has been recognized as a possible pathway in the development of OW/OB in some children [16, 32, 43], and is often a target in preventive interventions [11, 28, 31, 44–47].

The importance of helping children develop SREI was identified two decades ago [48]. The evidence base about SREI and ASR in childhood has substantially expanded in recent years, and has begun to draw on the constructs, evidence, theories and methodologies associated with GSR, especially constructs such as the neurocognitively oriented Executive Function (EF) [20, 21, 49–57] the temperament-based Effortful Control (EC) [12, 29, 58–60], Michel’s delay-of-gratification paradigm [24, 61–63], and emotion regulation/dysregulation [14, 26, 64]. However, despite some cross-fertilization, to date, there do not appear to have been efforts to systematically compare and contrast research and theory in what remain as relatively separate bodies of scholarship in ASR and GSR.

The purpose of the present narrative review is to conduct a reciprocal analysis of ASR and GSR in childhood through an examination of (1) key concepts and processes in GSR and ASR, (2) evidence about the possibility of common processes underpinning GSR and ASR, and (3) the extent to which GSR could be implicated in ASR-related outcomes such as disinhibited eating, Body Mass Index (BMI) and obesity. A reciprocal analysis implies that there are likely to be parallels as well as contrasts between ASR and GSR, with the potential for GSR to provide insights for ASR as well as ASR to provide insights for a wider treatment of GSR. The primary focus is on childhood, meaning from infancy to about ages 6 or 7. All areas included in the review themselves represent substantial bodies of knowledge. As a consequence, at times the coverage is somewhat introductory.

The evidence suggests that there are relatively unique features of ASR when compared to GSR. As a consequence, we argue there is value in an integration of research and theory about GSR and ASR, so that the broad field of self-regulation in childhood encompasses a number of domains including those related to both non-food self-regulation (e.g., emotions, actions and cognitions) and ASR. The discussion includes implications for research and theory that arise from linking GSR and ASR under a common self-regulation umbrella.

The international literature on weight gain and obesity has recognized that the overconsumption of foods and beverages, especially palatable and energy dense food, is an intractable problem in current obesogenic environments [31, 39, 41, 65, 66]. Drivers of food consumption are complex and multifactorial and cannot easily be solved with simple interventions. There is a need to better understand how healthy food consumption can be supported. Better knowledge of processes and mechanisms underlying individual differences in the development and disruption of appetite regulation and behavior could help to explain how and why individuals respond differently to food environments and to eating interventions. Even with a widespread application of policy levers to effect change in food environments, understanding the development of healthy self-regulation in children could contribute new information on possible solutions to the problem of achieving healthy food choices and intakes in obesogenic environments.

## Methods

To identify literature and relevant journals, we searched titles, abstracts and key words in databases (PubMed, PsycINFO, SCOPUS, Web of Science, Google Scholar) using the terms ‘self-regulation’, ‘appetite self-regulation’, or ‘self-regulation of energy intake’ together with associated constructs (e.g., Executive Function, Effortful Control, delay-of-gratification). We followed up with backward and forward snowballing. Given the quickly developing nature of the field, special efforts during the time of writing and up to submission were directed at “in press” articles from the key journals that were identified. Selection of articles was mainly limited to children or childhood, with a focus from infancy to age 6 or 7 years. The search also yielded literature from middle childhood, adolescence and adulthood and a selection of these publications was used to add insights about possible processes in GSR and ASR.

## Results

The results are presented first for key concepts and processes in general self-regulation followed by parallel literature on appetite self-regulation. Evidence pertaining to common underpinnings of GSR and ASR is presented. Finally, theory and evidence about links between food and non-food delay-of-gratification and ASR-related outcomes is examined.

### Key concepts and processes in general self-regulation

In developmental science, self-regulation is often conceived as a general goal-directed behavior, with self-control seen as an instance of successful self-regulation [3, 8, 67]. However, self-control and other concepts such as delay-of-gratification, temporal discounting, inhibitory control, executive functioning, effortful control, cognitive control “have all been related to, and are often treated as synonymous with, self-regulation” ([68] p. 91). At the broadest level, self-regulation/self-control has been conceived as involving an ability, capacity or use of strategies to override or change one’s inner responses such as desires or impulses, as well as to interrupt undesired behavioral tendencies and refrain from acting on them [69], “the process or behavior of overcoming a temptation or prepotent response in favor of a competing goal (either concurrent or longer term)” ([67] p. 80), or as executive processes modulating prepotent responses [68].

While there seems to be broad definitional agreement, a number of reviews have summarized differences in the core constructs, measures or definitions pertaining to GSR in childhood, such as EF, EC, self-regulation, inhibitory control and delay-of-gratification [3, 24, 68, 70]. In terms of the precise definitions of self-regulation and its central constructs, there can be considerable differences as shown by Cole et al.’s [68] table of selected theories and definitions of self-regulation, a parallel table by Nigg [3] and other critical analyses (e.g., [67]). Cole et al. ([68] p. 91) noted that the US National Institutes of Health “lamented the plethora of terms, conceptualizations, and methods used to define and study self-regulation”, arguing that this hindered our understanding of the basic mechanisms associated with important health and developmental outcomes.

Despite this complexity, there is some agreement about core constructs and processes in general self-regulation. Much of this agreement is captured in the reviews by Cole et al. [68] Gagne [24], Nigg [3] and Bridgett et al. [71], from which the core features of what Nigg [3] described as a domain-general model of self-regulation can be outlined. From these reviews and other literature, there appear to be four main elements of a domain-general model of self-regulation. First, the model involves recursive top-down and bottom-up processes in the regulation of action, emotion and delay of gratification. Top-down and bottom-up processes can be differentiated conceptually, but also at the level of neurobiology [72]. Top down processes have been described as “reflecting more effortful/executive control process served by cortical structures in the anterior cingulate cortex” ([71] p. 603). Bottom-up processes have been described as “reflecting more automatic (reactive) processes served primarily by subcortical structures” ([71] p. 603) and include spontaneous, emotion-driven appetitive processes. In addition to bottom-up processes being the targets of self-regulation, they can also be the source of self-regulation [3]. For example, fear of or avoidance of weight gain could contribute to regulation when palatable food is available.

Second, in a domain-general model, self-regulation is a dynamic process involving an interplay between top-down and bottom-up processes over time [68]. Third, there are two major elements of bottom-up processes, namely bottom-up avoidance (e.g., behavioral inhibition/fear, anxiety or behavioral over-control) and bottom-up approach (e.g., impulsivity/behavioral under-control) [3, 71]. Fourth, in a domain-general model, EF and EC are centrally implicated in top-down processes [3, 71].

The impetus for self-regulatory strategies often derives from reactive, spontaneous, appetitive or avoidant bottom-up processes. The power of bottom-up processes is reflected in the choice of Cole et al. [68] to describe these as “prepotent responses”. There is a substantial body of evidence in the literature on GSR (and as we show below, an essentially parallel literature about ASR) concerning the role of both behavioral inhibition/fear and impulsivity [3, 71]. Nevertheless, it is not a one-way process with the impetus for self-regulation always to be found in bottom up processes. As Nigg ([3] p. 365) noted, “top-down systems can activate, suppress, or bias bottom-up responses”. At the same time, top-down control is effortful, so that bottom-up processes can influence the nature and extent of action control [73].

As the terms “effortful”, “executive” and “control” imply in describing top-down processes, EF and EC pertain largely to voluntary, conscious, or deliberate cognitive processes in the regulation of behaviour. EC is a temperament-based construct and “is defined as the ability to inhibit a dominant (motor, vocal, emotional or cognitive) response and to activate a subdominant response” ([74] pp. 1-2). The components of EC include inhibitory control, planned action, effortful attention, conflict resolution, error correction and abilities to delay or wait [74, 75]. Temperament is assumed to involve genetically-based inherent characteristics and it has been reasoned that EC is an important underpinning of self-regulated behavior [18], and it has been a core aspect of research on emotion regulation [23, 76–79].

EF originated from the field of clinical neuropsychology and “refers to the more deliberate or goal-directed, top-down neurocognitive processes involved in self-regulation” ([74] p. 2). It has been considered to be a significant element of self-regulation [80]. Hendry et al. ([81] p. 2) characterized EF as involving “higher-order self-regulatory processes”. The processes include inhibitory control, cognitive flexibility and working memory [24, 74, 82–84]. Blair ([70] p. 3) said of EF that it involves “the ability to hold information in working memory, to inhibit fast and unthinking responses to stimulation, and to flexibly shift the focus of one’s mental frame, is more or less the foundation for the intentional, volitional self-directed control of behavior. The cognitive skills that make up this construct help us to limit impulsive responses, to regulate emotions, and to avoid bad decisions that might bring short-term gain but longer-term problems”. Inhibitory control is a component of both EC and EF. In the case of EC, it is conceived as a dimension of temperament, while in EF it is treated as a cognitive process. Both EC and EF contain components in addition to inhibitory control (e.g., effortful attention and conflict resolution in EC, and cognitive flexibility and working memory in EF), but for present purposes the emphasis is on inhibitory control as a core ingredient of self-regulation.

The inhibitory control component of EF has usually been assessed in childhood using behavioral or performance measures involving emotionally neutral tasks that require children to inhibit a dominant response, as assumed to be required in self-regulation [24, 85, 86]. Examples are the silly sounds Stroop task, where children are asked to make the sound of a dog to a picture of a cat and the sound of a dog to a picture of a cat, or the pencil tap task, where the child taps twice if the experiment taps once and the child taps once if the experimenter taps twice.

The assessment of EC in childhood, including inhibitory control, has been based on both parent-rated temperament for toddlers through childhood [24, 87, 88] and performance-based measures, especially for preschool-aged children [87, 89]. The latter have included comparable tasks to those used to measure inhibitory control under the umbrella of EF. For example, children saying circle when shown a square and vice-versa [89]. For present purposes, the main point to highlight about these inhibitory control tasks for both EF and EC is their emphasis on inhibiting salient responses and on top-down processes. As Lin et al. [74] argued, EC and EF have their origins in different historical disciplines, but they have a number of conceptual and empirical overlaps.

Considerable attention has been directed to the question of the empirical overlaps of EF and EC. Lin et al. [74] found that measures from the two domains could load on a single “self-regulation” factor. Commonality between EF and EC has been found via a “cognitive control” factor that included temperament-rated inhibitory control and performance on EF inhibitory control tasks [80]. Nevertheless, there is also evidence that behavioral measures of EF and self-report measures of EC in adolescents could predict academic performance in complementary and independent ways [83]. At least in adolescents, Zorza et al. [83] argued that EF and EC share some elements, but do not completely overlap. In preadolescents, Tiego et al. [90] found that behavioral ratings of EF and EC measured the same self-regulation construct. However, based on assessments at age 4 years, Backer-Grondahl et al. ([8] p. 2) argued that EF and EC are “not completely synonymous”. Lengua et al. [75] suggested some separation of the executive and delay components of EC, possibly because they could stem from different brain regions, have different developmental courses, predict and relate to adjustment differently, and relate differently to cortisol. In the latter case they speculate that executive, but not delay tasks could be related to cortisol. Overall, therefore, while there seems to be considerable commonality between EF and EC, especially in the measurement and role of inhibitory control, questions about the separation of the components, both within and between EF and EC remains somewhat open.

EF has been broadened beyond abstract and decontextualized tasks through a distinction between “hot” EF and “cool/cold” EF [53, 74, 86, 91, 92], with “hot” implicated in emotionally arousing, or rewarding situations (such as tasks involving rewards of snacks, toys or gifts and measures of wait times and self-control behaviors) and “cool” in emotionally neutral situations (such as the decontextualized and cognitive tasks above, where there are no rewards or punishers). The use of hot and cool EF draws on the hot/cool delay of gratification distinction earlier highlighted by Mischel and colleagues [93, 94]. So-called hot situations imply a greater role for bottom-up processes, while cool situations are more amenable to top-down cognitive processes and decision-making. The hot/cool distinction is especially relevant for a reciprocal analysis of GSR and ASR because of its centrality in both domains of self-regulation.

In relation to possible differences between hot and cool self-regulation, Zelazo and Carlson [92] argue that the neural systems supporting EF could vary as a function of the motivational significance of the situation. They also suggested that the development of hot EF might lag the development of cool EF, and that for children younger than about 6 years, the distinction between hot and cool EF is emerging, as part of the increasing specialization of neural systems. Gagne [24] argued that hot strategies involve emotional or reactive responses, while cool strategies draw on cognitive processes, with younger children more prone to be reactive than older children.

In seminal research, Mischel and colleagues investigated aspects of will-power and self-control in preschool children using the now well-established delay-of-gratification paradigm with food rewards, in what is often referred to as the “marshmallow” task [8, 62, 93, 95]. The wait or delay aspect of delay-of-gratification is also inherent in inhibitory control as conceived in both EF and EC and these aspects are now frequently used in the measurement of inhibitory control. In delay-of-gratification tasks, both food and non-food rewards (such as an appealingly wrapped gift) have been used [14, 19, 74, 96]. Because of the use of a reward in the delay-of-gratification paradigm it has often been used as a measure of hot self-regulation [8, 93].

#### Overview

Central in models and analyses of GSR is the notion of the recursive interplay between top-down and bottom-up processing. In the case of bottom-up, the emphasis has been on spontaneous or reactive responses that can be either approach or avoidant. Much attention has been directed to the top-down processes via research on the function of EF and EC, especially via the inhibitory control element. The dynamic interplay or mutual influence between top-down and bottom-up processes, including the potential for bottom-up processes to interfere with top-down regulation, has also been a feature of models of GSR [3, 68]. Nigg [3] referred to the self-regulation “universe” in presenting a glossary of major terms relating to self-regulation. Here we have introduced some features of that universe to facilitate a reciprocal analysis of GSR and ASR.

### Key concepts and processes in appetite self-regulation

The attention to overweight and obesity in children in recent decades has probably led to an emphasis on processes associated with ASR difficulties more than on the development of ASR in childhood. In contrast, in GSR there seems to have been a greater balance between attention to (1) the development of self-regulation and positive outcomes and (2) self-regulation difficulties or deficits. The greater positive emphasis in the GSR literature could arise from a more even focus on successful adjustment outcomes (e.g., in social, emotional, and academic domains) as well as maladjustment.

In general, the GSR description of self-regulation as pertaining to overriding inner responses, interrupting undesired tendencies and refraining from acting on them, or in terms of executive processes modulating prepotent responses, has also been generally applied to ASR (e.g., 30). In the case of ASR, the inner responses are internal signals about hunger and satiety and the prepotent responses are associated with food and eating. As already noted, research and theory about ASR implicates a wide range of influences and processes in self-regulation, many of which appear to be relatively unique to ASR.

Our survey of the literature showed that a large number of constructs has been used to characterize processes or to measure ASR in childhood, including ASR difficulties. The constructs are illustrated in Table 1 and extend across the two main sources of bottom-up processes identified in the literature on GSR, namely approach (such as food responsiveness, hedonic aspects of food, emotional eating), and avoidance (such as food avoidance, food neophobia and picky eating). The breadth and diversity of constructs that are relevant to processes and analyses of ASR in childhood is evident. The constructs in Table 1 have been grouped under the three main headings of bottom-up approach (e.g., food approach), bottom-up avoidant (e.g., food neophobia), and top-down. An additional grouping captures other constructs and those that seem to incorporate aspects of both top-down and bottom-up processes. The constructs in Table 1 cover research where ASR-related measures have been the dependent variable (such as parent influences on components of ASR, changes in ASR across development, and the heritability of ASR constructs), but also where they have been the independent variable (such as how parents respond to or perceive the components of ASR in children). The table also includes a number of reviews that discuss or examine a construct.

**Table 1:** An illustrative list of constructs applicable to ASR in childhood and their measurement

Construct	First author, publication year	Sample description/age focus for reviews	Construct Measurement	Study design	Results/selected findings
Bottom up (approach)					
Food responsiveness	Carnell (2016) [97]	4–5 year old UK children and mothers, mainly White British	CEBQ	Cross-sectional, with preloading and observed lunch intake plus parent reported eating behaviors	Higher food responsiveness was linked to greater total food intake
	Cross (2014) [98]	Low income African American and Hispanic parents and their preschool children aged 4 years	CEBQ	Cross-sectional survey of parent rated eating behaviors and parent feeding practices	Food responsiveness related to mothers’ restrictive feeding practices
Reward sensitivity/response to food and food cues	Adise (2019) [99]	7–11 year old US children and families, mainly White, middle income	fMRI	Cross-sectional using neuro imaging assessment of brain responses to food and money rewards	Higher food responsiveness linked to decreased brain responses to winning food rewards. Regions associated with reward, cognitive control and emotion may play a role in the brain’s responses to food
	Yokum (2019) [100]	US adolescents 14–17 years, 77.7% European American	fMRI	Longitudinal design measuring neural activity for gained weight versus weight stable groups	Suggest that initial hyper-responsivity to palatable high-fat food tastes could be related to future weight gain
	Shapiro (2019) [101]	From a pre-birth longitudinal cohort of US children, ethnically diverse. Tested at 4–6 years of age	fMRI	Cross-sectional with laboratory-based measurement of Eating in absence of hunger (EAH) and a brain scan	EAH was associated with activity in a major reward network, and reduced connectivity between brain regions associated with reward and those associated with response inhibition
Enjoyment of food	Carnell (2016) [97]	4–5 year old UK children and mothers, mainly White British	CEBQ	Cross-sectional, with preloading and observed lunch intake plus parent reported eating behaviors	Higher enjoyment of food was linked to greater total food intake
	Cross (2014) [98]	Low income African American and Hispanic parents and their preschool children aged 4 years	CEBQ	Cross-sectional survey of parent rated eating behaviors and parent feeding practices	Higher enjoyment of food was linked to more restrictive feeding practices in African American families
Hedonic/reward aspects of food and hunger	Alonso-Alonso (2015) [102]	Not age-based	Review	Examined the neuroscience of food reward	Discussed homeostatic and non-homeostatic (related to the brain’s reward system) influences on the regulation of food intake
	Lowe (2007) [103]	Not age-based	Review	Examined hedonic hunger as a new eating motive	Proposed a distinction between homeostatic and hedonic eating
Subliminal reward signals	de Araujo (2020) [104]	Not age-based	Review	Examined human and animal research about processes associated with food reward	Proposed a two-path model of food reward that included subliminal reward signals and conscious liking
Reward neurocircuitry	Reichelt (2015) [105]	Not age-based	Review	Examined neurocircuitry associated with the reinforcing value of foods and inhibitory control	Set out a model of food cue effects on homeostatic appetite signals and reward neurocircuitry
Emotional eating/over-eating	Lumeng (2014) [106]	Low-income (Head Start) US children aged 3–4 years and parents	CEBQ	Cross-sectional using parent questionnaires plus child weight and cortisol measures	Family stress was linked to overweight, with this mediated by emotional eating in boys
External eating	Jahnke (2008) [107]	German mothers of preschool children aged 3–6 years. Diverse SES	DEBQ	Cross-sectional using parent-questionnaires	Overweight children scored higher on external eating
Consumption of problematic foods	Jahnke (2008) [107]	German mothers of preschool children aged 3–6 years, diverse SES	Parent reports of child food consumption	Cross-sectional using parent questionnaires	Parent ratings showed that children with higher weight status ate significant less problematic food
Healthy food preferences	Anzman-Frasca (2018) [108]	Prenatal to early childhood	Review	Examined evidence about promoting healthy food preferences	Early exposure to healthy foods can support subsequent acceptance of these foods
	Russell (2016) [109]	Diverse sample of Australian preschool children aged 3–5 years and parents	Parent reports of food preferences	Cross-sectional, with measures of parent-reported child appetitive traits (CEBQ) and food preferences	Healthy food preferences were related to enjoyment of food, satiety responsiveness and fussiness
Eating in the absence of hunger (EAH)	Leung (2014) [58]	Low-income (Head Start) US preschool children and their caregivers, diverse in race and ethnicity	Observed EAH using the free access protocol	Cross-sectional with measures of parent-reports of temperament and obesogenic eating behaviors plus observed EAH	Higher temperamental surgency, but not effortful control, was related to more EAH
Impulsivity	Bennett (2016) [110]	UK parents (mainly tertiary educated) and their children aged 2–4 years	Parent ratings on ECBQ, and child impulsivity, plus laboratory assessments of child impulsivity	Cross-sectional, using parent questionnaires and laboratory measures	Girls high in trait-like impulsivity and boys high in motor impulsivity could be more prone to display food approach behaviors associated with weight gain when parents monitor their intake less.
Disinhibited eating	Shapiro (2019) [101]	From a pre-birth longitudinal cohort of US children, ethnically diverse. Tested at 4–6 years of age	Disinhibited eating measured using the EAH free access protocol	Cross-sectional with laboratory measurement of EAH and a brain scan	Provided new evidence of the neuronal correlates of disinhibited eating in young children
	Russell (2018) [29]	Childhood	Review	Narrative review of development of appetitive traits using insights from research and theory in developmental science	Outlined a biopsychosocial model of the development of appetitive traits, including disinhibited eating in childhood
Eating rate	Carnell (2007) [111]	UK children 4–5 years of age and parents (mainly mothers, White British and affluent)	Observed eating rate	Cross-sectional with observed eating behaviors plus parent-completed CEBQ	Faster eating was linked to higher food responsiveness and enjoyment of food. Slower eating was linked to higher satiety responsiveness
Bottom up (avoidance)					
Food neophobia/picky eating	Russell (2018) [29]	Childhood	Review	Narrative review of development of appetitive traits using insights from research and theory in developmental science	Outlined a biopsychosocial model of the development of appetitive traits, including food neophobia in childhood
	Cole (2017) [112]	Children less than 30 months of age	Review	Examined correlates of picky eating and food neophobia at different levels, for example, genetic, child, family, community	Highlighted the importance of investigating parent-child dyads and bidirectional feeding patterns
	Russell (2008) [113]	Population-based sample of Australian children 2–5 years and parents	CFNS	Cross-sectional, with measures of parent-reported food neophobia and food preferences	Food neophobia was negatively correlated with liking for all foods in the healthy food group of Australian Healthy Eating Guide
	Lumeng (2018) [114]	Low income US children and mothers. Entered study at 21 or 27 months of age.	CEBQ, BAMBI	Cross-lagged cohort questionnaire study at 21, 27 and 33 months of age	Concurrent association were found between picky eating and pressuring feeding, but no prospective associations
Food fussiness	Gregory (2010) [115]	Australian mothers of children 2–4 years mostly tertiary educated and Australian born	CEBQ	Cross-sectional using parent questionnaires about child eating behaviors, parent feeding, and concerns about child weight	Food fussiness predicted maternal pressure to eat, partially mediated by concern about child underweight
Food avoidance	Powell (2011) [116]	UK mothers of children 3–6 years, mostly White British	CEBQ	Cross-sectional with parent reports of parent feeding behaviors and child food avoidance	Maternal feeding practices significantly predicted child food avoidance
Emotional undereating	Bjorklund (2018) [117]	Representative community sample of Norwegian children 6–10 years and parents	CEBQ	Longitudinal with measures of child and contextual predictors of change in emotional over- and undereating	Lower family functioning at age 6 predicted emotional undereating at age 10
	Herle (2018) [118]	Subsample from Twins Early Development study at age 4 years, mainly White British	CEBQ	Cross-sectional with measures of genetic and environmental factors contributing to emotional over-and undereating	Genetic contributions to emotional undereating were not significant. Shared environmental factors explained 77% of the variance
Slowness in eating	Llewellyn (2010) [98]	Population-based sample of infant twins from England and Wales	BEBQ	Cross sectional heritability analysis of scales from BEBQ	Heritability was high for slowness in eating
Top down					
Delay-of-gratification	Lelakowski (2019) [72]	Diverse US sample of mothers, children aged 24–30 months	Snack delay task	Longitudinal, with measures of child temperament, parent feeding and child BMI	Impulsivity but not inhibitory control (snack delay task) was related to BMI
	Kidd (2013) [119]	US children aged 3–5 years	Marshmallow wait task	Cross-sectional with measures of children’s wait time and beliefs about environmental reliability	Wait time reflected differences in self-control and beliefs about the stability of the world
Reward/delay discounting	Bennett (2019) [120]	UK children aged 7–11 years and parents, mainly White middle class	Delay discounting task as a measure of impulsivity	Cross-sectional with measures of child impulsivity, adiposity, intake during a snack, and eating behaviors	Poorer performance on delay discounting was associated with greater snack intake
EC inhibitory control	Rollins (2014) [121]	US children aged 3–7 years and parents mainly White, middle to high income	CBQ	Short-term longitudinal with measures of restrictive feeding practices, intake of restricted food and child weight	Children with lower inhibitory control and higher approach showed greater increase in intake in association with experience of parental restriction
	Tan (2011) [122]	US parents with children 3–9 years	CBQ	Cross-sectional with measures of child self-regulation in eating, inhibitory control and parents’ feeding behavior	Self-regulation in eating was positively correlated with inhibitory control
EF inhibitory control	Fogel (2019) [123]	Children from an Asian cohort aged 6 years	Stop signal task as measure of inhibitory control	Cross-sectional with measures of child inhibitory control, eating behavior and adiposity	Lower inhibitory control was related to selecting larger food portion, multiple food servings and faster eating rates
	Shapiro (2019) [124]	From a pre-birth longitudinal cohort of US children, ethnically diverse. Tested at 4–6 years of age	Flanker task as measure of inhibitory control	Cross-sectional with measures of biomarkers of poor metabolic health and performance on cognitive tasks	Greater blood biomarkers of poor metabolic health were related to lower inhibitory control
Others/both top-down and bottom-up					
Homeostatic and hedonic systems cross-talk	Higgs (2017) [125]	Not age-based	Review	Examined evidence about the integration of metabolic, reward and cognitive processes in appetite control	Favors a framework that emphasizes cross-talk between the neurochemical substrates of hedonic and homeostatic systems
	Berthoud (2017) [126]	Not age-based	Review	Examined hedonic and homeostatic controls in the regulation of body weight	Presents neural models of the interaction between homeostatic and hedonic controls
Interoception	Keller (2018) [127]	Children	Review	Examined the role of the brain in children’s food choice and eating behavior, including brain regions associated with interoception	Noted findings suggesting a reduced awareness of internal homeostatic cues among individuals prone to obesity
Alliesthesia	Higgs (2017) [125]	Not age-based	Review	Examined evidence about the integration of metabolic, reward and cognitive processes in appetite control	Discussed alliesthesia: food is more liked when hungry, less so when eating when full. Noted associations with decreases in reward-related brain activations
	Berridge (2010) [128]	Not age-based	Review	Examined brain mechanisms associated with obesity or eating disorders, including alliesthesia	Suggested possible brain-based mechanisms for hunger increasing “liking” and “wanting” food
Caloric compensation	Carnell (2007) [111]	UK children 4–5 years of age and parents, (mainly mothers, White British and affluent)	Observed using preload protocol	Cross-sectional with measures of children’s ability to regulate intake depending on the caloric content of a preload plus parent-completed CEBQ	Higher satiety responsiveness (CEBQ) was associated with better average caloric compensation
Compensation for energy density	Brugaileres (2019) [129]	French infants at 11 and 15 months of age and mothers	Observed using preload protocol	Short-term longitudinal with measures of changes in adjustment of intake to energy density	At both ages, infants undercompensated for the energy of the preload. Compensation ability decreased from 11 to 15 months. The greater the decrease, the higher weight status at 2 years of age
	Johnson (2000) [28]	High SES US children 4–5 years of age and parents	Preload protocol	Short-term longitudinal intervention to help children recognize cues of satiety and hunger to compensate for energy density	Large individual differences in self-regulation at baseline. The intervention improved children’s self-regulation
Compensation across meals and over days	Leahy (2008) [130]	US children 3–5 years of age. Parents mostly White with a university degree	Varied energy density of prepared meals	Short-term longitudinal with measures of intake in response to differences in energy density over 2 days using a cross-over design	A decrease in energy density led to a decrease in energy intake; children did not compensate in their energy intake (calories) according to the energy density of the meals
Food choice/processed food effects	Small (2019) [131]	Not age-based	Review	Examined two systems driving food choice: metabolic signals about nutritional content, and conscious perceptions e.g., about flavor, caloric content, healthfulness	Argues there is evidence that nutritional signals about processed food are not accurately conveyed to the brain
Food “liking” and “wanting”	Keller (2018) [127]	Children	Review	Examined the role of the brain in children’s food choice and eating behavior, including the neural drivers of food “liking” and “wanting”	Summarizes evidence about the neural drivers of affective response to food (“liking”) and the incentive salience of food (“wanting”)
	Berridge (2016) [132]	Not age-based	Review	Examined brain mechanisms associated with “wanting” a reward (including food) and “liking” the same reward	Addiction could be associated with excessive amplification of “wanting”, especially triggered by cues about anticipated rewards and pleasure. Heightened dopamine reactivity such as stress and emotions could increase “wanting”
Satiety responsiveness	Carnell (2016) [97]	4–5 year old UK children and mothers, mainly White British	CEBQ	Cross-sectional, with preloading and observed lunch intake plus parent-reported eating behaviors	Higher satiety responsiveness was linked to lower total food intake
	Cross (2014) [98]	Low income African American and Hispanic parents and their preschool children	CEBQ	Cross-sectional using measures of parent-rated child eating behaviors and parent-reported feeding practices	Higher satiety responsiveness was associated with greater pressure to eat in African American families
Satiation and satiety	Blundell (2010) [133]	Not age-based	Review	Examined specific measures of satiation, satiety, hunger and food consumption, including “liking” and “wanting”	Sets out a model of the impact of foods on satiation and satiety. Discussed approaches to the measurement of satiation and satiety
	Bellisle (2012) [134]	Not age-based	Review	Examines the satiating power of foods with sweeteners. Included “liking” and “wanting” and their role	Highlighted methodological challenges in measuring satiation and satiety

*CEBQ* Children’s Eating Behaviour Questionnaire, *BEBQ* Baby Eating Behaviour Questionnaire, *TMCQ* Temperament in Middle Childhood Questionnaire, *ECBQ* Early Childhood Behaviour Questionnaire, *CFNS* Child Food Neophobia Scale, *BAMBI* Brief Autism Mealtime Behaviour Inventory, *CBQ* Children’s Behavior Questionnaire, *EF* Executive Function, *EC* Effortful Control

The list in Table 1 reveals a wide variety of constructs that appear to be particular to ASR rather than to GSR. It seems noteworthy that homeostasis is frequently drawn on in analyses not only of appetite regulation, but also in appetite self-regulation. It is illustrated by the idea that rather than independent homeostatic and hedonic systems, there is an interaction between the neurochemical substrates of the two systems in the regulation of energy intake and weight. This has been described as involving “crosstalk” between the homeostatic and hedonic systems [125, 126, 135]. The inclusion of homeostasis in approaches to ASR seems to provide a contrast with GSR, where Nigg [3] excluded allostatic and homeostatic processes in his review of self-regulation. The constructs included in Table 1 point to the complexity and range of different components implicated in ASR.

Approaches to measurement in ASR (some referred to in Table 1) have covered three overlapping areas relating to both self-regulation and self-regulation difficulties: (1) processes, (2) individual differences and (3) regulatory skills or strategies. In the case of processes, there has been considerable use of functional magnetic resonance imaging (fMRI). For example, fMRI has been used to assess increased responsivity to palatable food receipt and cues, possible inhibitory control activation to suppress urges to consume palatable food, and reduced fat taste sensitivity that may impair satiety responses [100] as well as the origins and role of sweet taste in reward-based eating in children [136] and stress-induced cortisol effects on reward sensitivity and appetite regions of the brain [137].

In the measurement of individual differences in eating behaviors or appetitive traits in childhood, both neural and behavioural measures have been used [138]. Parent ratings of appetitive traits have been widely used. This has especially involved use of the Children’s Eating Behaviour Questionnaire (CEBQ) [139, 140] the parallel Baby Eating Behaviour Questionnaire [140, 141] and other questionnaires, such as the Child Food Neophobia Scale [142]. The CEBQ has two broad dimensions of food approach and food avoidance (thus matching the two main aspects of bottom-up processes). Individual CEBQ scales include food responsiveness, satiety responsiveness and food fussiness.

In single-session experimental and/or naturalistic contexts, the preload protocol and observations have been used to measure calorie compensation, eating in the absence of hunger, i.e., eating beyond satiation, and eating rate [28, 52, 97, 129, 143–147]. A similar strategy has been used to measure energy intake and compensation over several days [130]. Results of these measurement approaches are used either to study individual differences in ASR or as measures of self-regulatory skills (e.g., the ability to compensate for preload) or self-regulatory difficulties (e.g., eating in the absence of hunger). The widely used delay-of-gratification paradigm discussed below can also be treated as an approach to measure skills in self-regulation, or to measure individual differences. Measures of EF, EC and emotion regulation have been incorporated into research on ASR and related to child weight status [52].

Focused models and conceptions of ASR (especially as they relate to self-regulation difficulties or deficits that seem to be associated with disinhibited eating) [31–34, 101, 148–150] include at least top-down regulation, hedonic aspects of food including sweet taste preferences [151], biological processes in the control of appetite [33, 34], as well as social, physical and macro-level environments, and psychological and neural control mechanisms.

The idea of bottom-up and top-down processes and their interplay intrinsic in approaches to GSR has been part of recent conceptualizations of ASR [32, 39, 44, 61, 72, 101]. While in the case of ASR, the self-regulatory processes include top-down, cognitive and decision-making processes, regulation is also dependent on body and brain responses (such as hormonal and brain signals about satiation) to the food, processes such as alliesthesia and interactions between metabolic, reward, and cognitive processes [125]. Consciously perceptible hedonic qualities of food could play a mostly transient role in food reinforcement, with a greater contribution from subliminal gut-brain reward pathways [104]. Further, homeostatic (i.e. regulatory) and hedonic systems in relation to food have evolved to communicate and influence one another [125, 152] and there is a complex interplay of homeostatic and non-homeostatic controls [102]. Cognitive and behavioral aspects of ASR, involving social and goal-directed behaviors and decision-making involved in top-down processes [32], might interact with neural and endocrinal substrates. The interplay between bottom-up and top-down processes in ASR is reflected in the argument that appetite self-regulation occurs “at cognitive, emotional, motivational, biological, and behavioral levels” ([11], p. 71).

Cognitive processes involved in appetite regulation have been separated in terms of those operating before a meal and those during a meal [125]. In a similar way, Smethers and colleagues [153, 154] argued that there are different possible time courses in relation to ASR. One possibility is a particular eating or feeding episode which is affected by a range of factors, some of them which are unique to eating such as sensory specific satiety and food variety [155, 156]. Another relates to compensation or adjustment from a meal or snack to the next opportunity for eating, or over several days. For example, preschool children have been found not to adjust energy intake in response to energy density or portion size over a 5-day period [153, 154]. There is control of immediate gratification versus control of overall energy balance [157]. Different time courses seem to imply different bottom-up and top-down processes.

ASR also covers both the quantity and composition of dietary intake [48]. This means that ASR involves not only the regulation of energy intake, but also the choice of foods, especially with respect to “healthy” and “unhealthy” diet and food choices [34, 35, 158–162]. Finally, in the case of ASR, bottom-up processes can arise in different ways, such as from hunger (and the food could be healthy or unhealthy) versus from the attraction of palatable food, or the sweet taste [136, 151], from attraction arising from food having been restricted [163, 164], especially for children lower in inhibitory control [121], the desire to eat to regulate emotions [29, 118, 165, 166], and in response to stress [167–170]. This means that what is being responded to and what or how top-down processes might be drawn on seems variable and uncertain in the case of food and eating: is the same top-down process required in relation to self-regulating responses to foods of large portion size and foods higher in energy density, for example? And does this differ according to emotional state and hunger levels?

### Evidence about common underpinnings of GSR and ASR and contributions to appetitive traits and BMI

The above comparison of GSR in developmental science and ASR highlighted possible differences between them, but also a number of parallels or overlaps. This raises the question of what the evidence indicates about whether and how GSR and ASR have common underpinnings, especially with respect to EF, EC and delay-of-gratification. The evidence reviewed above shows the importance of EF, EC and delay-of-gratification in GSR. The question is whether there is similar evidence about ASR. In addition, a linked question arises from research on delay-of-gratification. Here the question is whether and how food and non-food related delay-of-gratification is associated with children’s appetitive traits, weight and obesity. If food and non-food delay-of-gratification are associated with ASR-related outcomes such as disinhibited eating and obesity in similar ways, this could suggest a close linkage between GSR and ASR. On both questions the evidence is complex and diverse.

On the positive side, there is some evidence and claims that elements of EC contribute to ASR-related outcomes such as children’s eating behavior, weight, weight gain, or nutrition risks [21, 56, 60, 122]. This research has included preschool children [56, 60]. There is parallel evidence and claims that components of EF are also associated with ASR-related outcomes [21, 49, 50, 53, 55, 56, 171]. Much of this evidence seems to involve children of elementary school age or older [53, 55, 171–173]. There seems to be limited evidence about EF and ASR-related outcomes in younger children. Nevertheless, a recent study [49] reported that change in EF from 3 to 5 years was inversely related to change in BMI from age 2 to 5 years.

Positive results for EF and/or EC have not always been found in the literature on ASR with young children. Hughes et al. [52] used a battery of eating and non-eating self-regulation procedures and measures with a sample of Hispanic preschool children and their parents. The battery of non-eating related measures was not significantly related to child BMIz. The battery included EF tasks, EC from parent reports, delay-of-gratification with a food reward and delay-of-gratification with a non-food gift reward. In a sample of 3 to 6 year-old children, Pieper & Laugero [56] found that parent reported inhibitory control was associated with parent-reported emotional over eating, but not to observed eating in the absence of hunger. Three EF tasks (including delay-of-gratification) also were not related to observed eating behavior (but parent-reported emotional eating was related to an EF selective attention task). In a meta-analysis of research with mainly adolescents and adults, Yang et al. [57] found that broad impairments on executive function were linked to obesity but only deficits in the inhibition and working memory components of EF were found for overweight participants. Tan and Holub [122] found that parent reported inhibitory control was related to parent-reported self-regulation in eating in a sample of children aged 3 to 9 years, but not to child BMI percentile. In research with preschoolers and their caregivers, Leung et al. [58] reported no significant relationships between caregiver-reported effortful control and six measures of caregiver-reported obesogenic appetitive traits.

#### Evidence about contributions of food and non-food delay-of-gratification to appetitive traits and BMI

In addition, investigations of relationships between EC and EF and children’s eating behavior and weight/weight gain, there has been a body of research based on measures of delay-of-gratification. The evidence here mainly pertains to (1) whether or not BMI, obesity or weight gain is predicted by delay-of-gratification for food and non-food tasks, and (2) differences between OW/OB versus normal weight children on delay-of-gratification tasks. The findings are partly complicated by the tendency for research to claim to be investigating “self-regulation” but use food as the reward in the delay-of-gratification procedure [43], despite questions about the comparability of food and non-food rewards as the evidence below indicates.

There are findings that performance with non-food rewards in delay-of-gratification can predict ASR-related outcomes, as well as other data suggesting that only food rewards are associated with ASR-related outcomes. In the former case, Saltzman and colleagues [20] argued that general self-regulation and ASR are “strongly related”, to the extent that a pathway to ASR is via GSR. In the end, this claim relied on the results of Graziano and colleagues [95, 174]. They used delay-of-gratification for an “appealingly” gift-wrapped box at 2 years as a measure of self-regulation/inhibitory control/reward sensitivity and compared overweight/at risk versus normal weight children at age 5.5 years [96]. Overweight/at risk children were in the 85th BMIz percentile or greater. They found inhibitory control (delay-of-gratification) was significantly related to BMI at age 2. In a further follow-up at age 10 years [174] they reported that BMIz-scores and changes in BMIz-scores from 4 to 10 years were related to “self-regulation skills” at age 2. In this case “self-regulation skills” was a single factor score combining laboratory measures of sustained attention, emotion regulation and delay-of-gratification using an appealing gift-wrapped box.

In speculating about the possible mechanisms linking “self-regulation deficits” to BMI and the development of weight problems 8 years later, they suggested that it could arise from “oversensitivity to novel and pleasurable activities” (p. 941) that could be present prior to the development of obesity, but were unsure about how this could contribute to obesity. Possibilities they proposed were that it could interfere with satiety processes such as recognizing the signals of satiety and stopping eating, and could contribute to unhealthy food preferences, both of which are elements of ASR. Francis and Susman’s [157] results also suggest that performance on delay-of-gratification (they called it a “waiting game” for a toy) at 3 years was related to weight gain in children to 12 years of age. A subsequent delay-of-gratification measure using food-reward at age 5 enhanced the prediction of weight gain.

When delay-of-gratification research has directly compared food and non-food rewards, there are a number of results pointing to the independence of performance on food and non-food tasks. Miller et al. [14] measured BMI and observed toddler (mean age 33.1 months) “self-regulation” responses in food (delay of gratification for a snack) and non-food (delay of gratification for a gift) tasks as well as emotional self-regulation from negative affect in frustration eliciting tasks (one food-related- no-touch cookie tasks and one non-food related-no-touch toy task). They reported that the ability to wait in the food delay task (but not in the non-food delay task) was associated with lower concurrent child weight (BMIz) and lower odds of overweight/obese status. They suggest that food responsivity could be implicated. Better emotional self-regulation in both tasks (less prone to distress in the face of frustration) was related to lower odds of overweight/obesity. A possibility the authors raised was that parents could engage in emotional feeding as a soothing strategy for children prone to negative emotions. As noted below, emotional feeding could be a factor in the disruption of ASR in childhood.

Obese and non-obese children from early childhood and middle childhood have been found to differ on delay-of-gratification tasks where food is a reward, but not when there are non-food rewards [175, 176]. These results are consistent with the suggestion ([177] p. 411) that “children who were overweight were particularly ineffective at inhibiting their responses towards food stimuli” (p. 411). They suggested that overweight children could be especially responsive to food cues and find it difficult to inhibit responses to those cues. This possibility is consistent with a finding that performance on delay-of-gratification with a food reward at age 4 predicted BMI 30 years later [178]. These authors suggested that this result could reflect executive function abilities. It needs to be noted, however, that performance on food-reward delay-of-gratification is not always related to ASR-related outcomes. For example, in a toddler.

sample, Lelakowska et al. [72] found that performance on the snack delay task at 24 months was not related to either parent-reported emotional overeating or child BMI at 30 months.

## Discussion

The present reciprocal analysis pointed to the potential for the integration of theory and evidence about GSR and ASR. Nigg [3] argued that self-regulation should be treated as domain general, and then specific terms used in relation to separate domains. For example, self-regulation of emotions, self-regulation of actions and self-regulation of cognitions. Calkins and colleagues [22, 23] have advanced similar arguments. Following this reasoning, the present analysis suggests that self-regulation of appetite should be added as a domain under the umbrella of self-regulation in childhood.

Overall, the present reciprocal analysis (an overview provided in Table 2) noted important parallels between ASR and GSR in childhood in terms of key concepts and possible underpinning processes, but, equally, ASR, like each of the other identified self-regulation domains of GSR, seems to involve a number of unique components and processes. Some of these are highlighted in Table 2. The evidence did not seem to support a conceptualization of GSR as providing a pathway to ASR. There were suggestions of common underpinnings, but the development of GSR and ASR in childhood is no doubt shaped by processes partly inherent to each domain and develop somewhat independently [52], but with increasing integration across childhood.

**Table 2:** Results overview

Domains	Food self-regulation (eg. ASR, SREI)	Non-food self-regulation (eg of action, emotions, cognitions)
Possible underpinning processes	EF, EC, Inhibitory Control, delay-of-gratification, recursive bottom-up and top-down processes	EF, EC, Inhibitory Control, delay-of-gratification, recursive bottom-up and top-down processes, Hot and cool/cold EF
Possible unique components and processes	Early homeostatic regulation, satiation versus satiety, hormonal and brain responses to food, different time courses of regulation (e.g., meal versus diet), regulation of quantity versus quality of diet, different influences on bottom-up processes (such as hunger versus palatable food signals), role of disinhibited eating in the disruption of ASR, and quantity and quality of food intake effects on brain development and EF.	Unique components and processes in non-food self-regulation
Relationships between food and non-food self-regulation	The pathway to food SR is via non-food SR (little evidence in support).	
	Food and non-food SR have common EF and EC underpinnings (limited evidence in young children, but support in middle childhood and beyond).	
	Non-food SR contributes directly to ASR-related outcomes such as BMI and obesity (little evidence in support).	
	Integration of aspects of non-food and food SR and the associated underpinning processes across childhood (likely and warrants further research).	

The relatively unique aspects of ASR seem to have parallels in the case of emotion regulation/dysregulation, at least with respect to neurobiological and endocrinal processes, as well as in terms of reading internal and external cues, and unique cognitive and behavioral processes [179–183]. In the case of self-regulation arising from fear/inhibition and impulsivity/disinhibition, unique features are also likely to be evident, especially in relation to demands placed on top-down processes in the regulation of cognition and action.

It is appealing to assume that comparable processes, abilities or capacities such as those inherent in EF, EC and in delay-of-gratification underpin both GSR and ASR. However, the evidence seems to be mixed for EF and EC, at least over the period from infancy to age 5 or 6 years of age. In relation to abilities associated with delay-of-gratification procedures, the evidence mainly points to differences for food and non-food related delays. In relation to ASR, there is also a question of the direction-of-effect [57, 171]. There is evidence about the impact of adiposity on the structure and function of the prefrontal cortex (which is linked to executive control processes), so that excessive consumption of appetitive calorie-dense food could contribute to impairments in executive function and then to reduced food self-regulation [150]. There is also evidence that metabolic health could impact cognitive function in preschool children [124]. Finally, both weight gain and impairments in executive function could be related to a common third factor such as stress [168, 184, 185], or genetic predispositions [171].

Collectively, processes in ASR seem to incorporate biological (e.g., bottom-up approach and avoidance), psychological (e.g., top-down inhibitory control) and social factors (e.g., external food and eating cues, and social contexts that impact both bottom-up and top-down processes). In this way, ASR appears amenable to a biopsychosocial approach in common with much developmental theory [186, 187] and in parallel with the development of appetite traits [29, 188] and overweight/obesity in childhood [16].

It is helpful to place the possibility of limited contributions of EF and EC to ASR-related outcomes in young children in a developmental context. It could be that with respect to food and eating, “children’s top-down control capacities are relatively immature compared to bottom-up processes” ([189] p. 111), where the hedonic value of food and early taste preferences contribute to the potency of bottom-up processes, and this could account for some of the results for preschool children. As we noted, however, there are consistent findings about the role of EF and EC in older children, adolescents and adults, for example, as suggested by meta-analyses [57] and literature reviews [171, 172].

Inherent in the conceptualization of self-regulation in childhood as occurring in different domains or levels, is the notion that the domains or levels build on and integrate over the course of development [3, 4, 22–24, 190]. For instance, Nigg [3] posited that “aspects of the SR universe can be organized hierarchically in relation to granularity, development, and time. Low-level components assemble into high-level components” (p. 361): “low-level operations like response inhibition and working memory support emergence of more complex operations like higher order EF” (p. 374), in a cascade type process. Nigg went on to argue that bottom-up processes emerge and mature earlier than top-down processes and that “different aspects of SR mature at different rates within bottom-up and top-down domains” (p. 375).

It seems that in developmental science research, more attention has been directed to top-down components of self-regulation than in work on ASR, as suggested at least by the body of research on the early development and precursors of EF and EC in non-food self-regulation [4, 24, 81, 89, 191, 192]. There seems to be scope for attention to similar questions about early precursors and their integration across childhood in the development of ASR. For example, whether or how early homeostatic energy regulation in infancy is linked to the emergence of different aspects of ASR in childhood. Increased research is needed on the contribution of the various dimensions of EF and EC to appetitive traits (e.g., responsiveness to food cues) across childhood. Equally, there is a need for more research on the nature and role of top-down and bottom-up processes in children’s eating and weight (e.g., 72), with attention to the elements of bottom-up processes and top-down processes, as well as how they interact, and changes in their role and interaction across childhood for different elements of ASR (such as making healthy food choices, resisting palatable foods, decisions about when to eat, decisions about stopping eating etc). Theory and evidence from GSR seem to have the potential to provide conceptual and methodological insights for approaches to ASR in these areas.

At the same time, scholarship on ASR has the potential to enrich knowledge and insights about general or non-food self-regulation in childhood. For example, by attention to possible parallels in the nature of hedonic and avoidant responses to internal or external stimuli (such as arise in relation to taste/food preferences, hunger and food), as well as the role of metabolic processes and brain structures and responses (such as arise in food-related situations). The case of ASR also suggests there is potential in adding ideas and analysis about what Cole et al. [68] referred to as Executive Processes, which they indicated involve attention, memory, reasoning and conscious decision making. For example, different executive process strategies could be implicated in responding to hunger signals versus palatable food signals and in relation to the initiation of eating versus the control of an eating episode [148] as well as to the broader control of food choice and diet. Comparable differences in executive processes might apply to non-food self-regulatory situations (such as more internally generated anger, versus external provocation, and when and how to stop an anger outburst or acting out episode).

An implication of drawing ASR under a common umbrella of self-regulation in childhood is that it generates a cross-fertilization of areas of research that have the potential, in turn, to advance knowledge about both GSR and ASR. New areas of research endeavor might include: 1) research and theory about the changing roles of top-down and bottom up processes across childhood as a function of different food and non-food regulatory situations 2) a greater integration of research and theory about the role of stress in the disruption of GSR and ASR, 3) more joint attention to situations or factors that increase (or decrease) the capacity and role of top-down and bottom-up process such as arises from visceral reactions to hyperpalatable and rewarding food cues [150], 4) expanding research on hot EF [193] to include various food-related contexts, 5) more research on when, why and how results differ for food and non-food related delay-of-gratification, 6) more attention to how the separate domains or levels of self-regulation (now including ASR) become integrated across childhood, 7) research on how homeostatic processes in energy intake and expenditure link with EF and EC, and 8) research on whether and how GSR might have parallels with food satiety and satiation.

## Conclusion

The present review suggests there are some overlaps between GSR and ASR: there is commonality with respect to the overall meaning of self-regulation, in the application of constructs such as EF, EC and delay-of-gratification, and in the utilization of bottom-up and top-down processes. The overlap is shown by the relevance for both GSR and ASR of the four main elements of the domain-general model outlined in the section on key concepts and processes in general self-regulation. But, it is also evident that ASR implicates factors that seem to be unique to food-related self-regulation. Consistent with similar arguments about GSR, it is reasonable to say that there is not yet a unified definition or model of ASR in childhood. This could serve as an impediment to research and theory development about the role of ASR in OW/OB in childhood.

The recognized domains of self-regulation in childhood include, emotions, actions and cognitions. Each of these bring somewhat unique features to questions about processes in the development and functioning of self-regulation in childhood. Clearly, also with some unique features, a case can be made to include ASR as a domain under the umbrella of self-regulation in childhood. This has the potential to enrich theory and research and serve as a significant heuristic for future scholarship about self-regulation in childhood.

## Data Availability

Not applicable.

## Abbreviations

ASR: Appetite self-regulation
BMI: Body Mass Index
EC: Effortful Control
EF: Executive Function
GSR: General self-regulation
OW/OB: Overweight/Obese
SREI: Self-regulation of energy intake
